# JNS Turns Five; Toddler No More

**DOI:** 10.21699/jns.v6i1.521

**Published:** 2017-01-01

**Authors:** Yogesh Kumar Sarin

**Affiliations:** Chief Editor, Journal of Neonatal Surgery

It has been a busy 5 years. So, on behalf of Emeritus Editor-in-chief Dr. Afzal Sheikh and my co-patron and Editor Dr. Bilal Mirza, let me take just a moment to celebrate and reflect.

Five years may be the blink of an eye in the print publishing world, but it’s practically a lifetime online. At this juncture, we take time out to reflect briefly on what has been, and comment on future directions.

As patrons and editors, I and Dr. Mirza had to work on few very vital issues. First and foremost concern was that it should not be looked upon an isolated Indo-Pak venture! Though in the first five years, 2/3rd of the contributions are from these two neighboring countries, the other 1/3rd valuable share comes from all parts of the globe (Table 1 and Fig. 1). We have an international reach now. About the annual viewership statistics, the number of annual visitor have risen by 1/3rd, the pageviews have risen 250%. However, the percentage of returning visitors has not risen that substantially, only from to 16.9% [1] to 26.6% over 4 years. We have better exposure after being indexed with plethora of indexing agencies including PubMed and PubMed Central.

**Figure:**
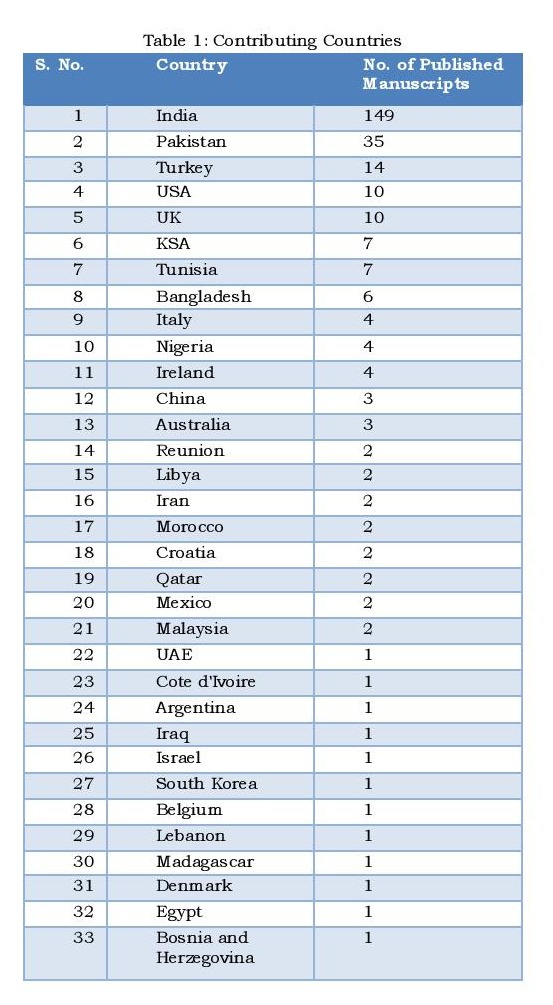
Table 1: Contributing Countries

**Figure:**
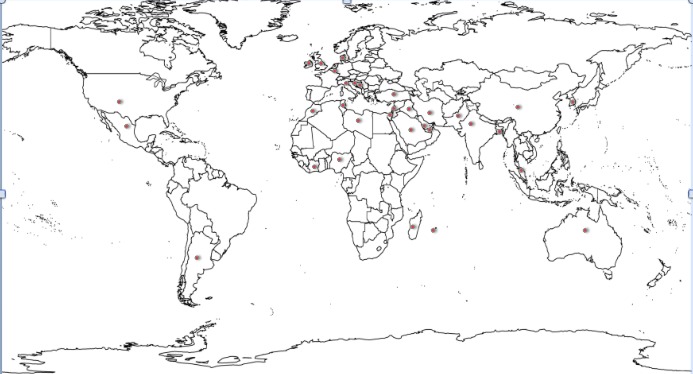
Figure 1: Origin of authorship* (* Red dots depict countries from where published manuscripts were received.)

On the first and second anniversaries of JNS, we had shared the top five manuscripts that had highest viewership in the year 2013 [2, 3]. On the fifth anniversary, we share here a simple and straightforward way to help gauge the productivity of a scholar. The following 6 manuscripts had more than 5 citations in international journals:

**Figure:**
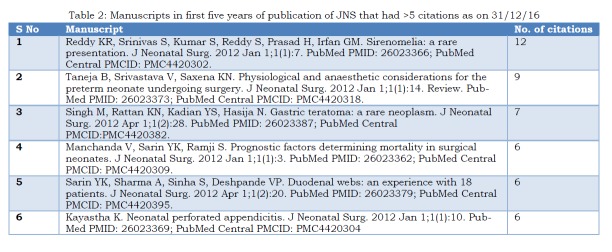
Table 2: Manuscripts in first five years of publication of JNS that had more than 5 citations as on 31/12/16

We congratulate the team of authors from Niloufer Hospital, Institute of Child Health, Osmania Medical College, Hyderabad, Telangana, India whose case report has scored 12 citations till date; kudos for another addition to i10-index [4].

Journal of Neonatal Surgery has got 266 citations since its inception for 284 citable items. I am not unduly perturbed with the low 2-year impact factors of the journal for the last 3 years (Fig. 2); the reasons are rather obvious. One, globally the surgical journals usually have far lower impact factors as compared to basic or clinical medical research journals. Two, we do not represent any official journal of any Medical Association, thus keeping the politics of the academicians away. We shall strive to go higher solely on the strength of its diverse and high-quality content. We invite material on the latest innovations and developments that are happening in the developed world. If you value the benefits brought by publishing in a high quality open access journal, with a dedicated editorial team, and a rigorous but fair and friendly peer review service, we look forward to receiving your next submission to JNS.

**Figure:**
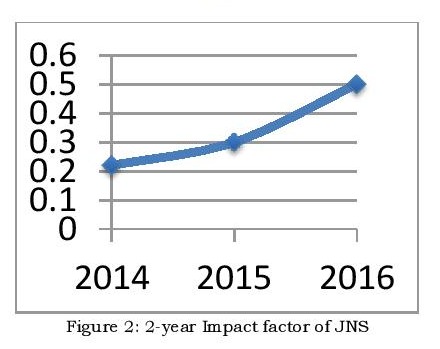
Figure 2: 2-year Impact factor of JNS

I close with once again expressing my sincere gratitude to our reviewers for their valuable time, as well as to our authors and the Journal’s Editorial Board for their most valuable support.

## Footnotes

**Source of Support:** Nil

**Conflict of Interest:** None
